# Surface Modification of Ammonium Polyphosphate for Enhancing Flame-Retardant Properties of Thermoplastic Polyurethane

**DOI:** 10.3390/ma15061990

**Published:** 2022-03-08

**Authors:** Zhiwen Wang, Yan Jiang, Xiaomei Yang, Junhuan Zhao, Wanlu Fu, Na Wang, De-Yi Wang

**Affiliations:** 1 Liaoning Provincial Key Laboratory for Preparation and Application of Special Functional Materials, Shenyang University of Chemical Technology, Shenyang 110142, China; zhiwenwang6018@126.com (Z.W.); na_jiangyan@sina.com (Y.J.); 2 Shenyang Research Institute of Industrial Technology for Advanced Coating Materials, Shenyang 110142, China; 13464038746@163.com; 3 Zhejiang Ruico New Material Co., Ltd., Huzhou 313018, China; xiaomei_yang@126.com (X.Y.); ruigaonm@126.com (J.Z.); 4 IMDEA Materials Institute, C/Eric Kandel, 2, Getafe, 28906 Madrid, Spain; 5 Escuela Politécnica Superior, Universidad Francisco de Vitoria, Ctra. Pozuelo-Majadahonda Km 1800, Pozuelo de Alarcón, 28223 Madrid, Spain

**Keywords:** thermoplastic polyurethane (TPU), flame-retardant, ammonium polyphosphate (APP), polydopamine (PDA)

## Abstract

Currently, the development of efficient and environmentally friendly flame-retardant thermoplastic polyurethane (TPU) composite materials has caused extensive research. Ammonium polyphosphate (APP) is used as a general intumescent flame retardant to improve the flame retardancy of TPU. In this paper, we developed a functionalized APP flame retardant (APP-Cu@PDA). Adding only 5 wt% of APP-Cu@PDA into TPU can significantly improve the flame-retardant’s performance of the composite material, reflected by a high LOI value of 28% with a UL-94 test of V-0 rating. Compared with pure TPU, the peak heat release rate, total heat release, peak smoke release rate, and total smoke release were reduced by 82%, 25%, 50%, and 29%, respectively. The improvements on the flame-retardant properties of the TPU/5%APP-Cu@PDA composites were due to the following explanations: Cu^2+^-chelated PDA has a certain catalytic effect on the carbonization process, which can promote the formation of complete carbon layers and hinder the transfer of heat and oxygen. In addition, after adding 5% APP-Cu@PDA, the tensile strength and elongation at the break of TPU composites did not decrease significantly. In summary, we developed a new flame-retardant APP-Cu@PDA, which has better flame-retardant properties than many reported TPU composites, and its preparation process is simple and environmentally friendly. This process can be applied to the industrial production of flame retardants in the future.

## 1. Introduction

As the most versatile engineering thermoplastic, thermoplastic polyurethane (TPU) has excellent tensile strength, good shock absorption performance, high wear resistance, oil resistance, water resistance, and other excellent physical and chemical properties [1]. Because of its outstanding performance, TPU is widely used in the fields of medicine and health, cables and wires, electronics and aerospace, etc. [2]. However, TPU is flammable, which severely limits its application due to fire-resistance requirements. Therefore, research on improving flame retardancy and reducing the release of toxic gases and smoke of TPU has attracted a lot of attention [3,4,5]. In recent decades, many flame-retardant materials have been extensively studied. These studies mainly include halogenated flame retardants, metal hydroxides, and compounds containing phosphorus, nitrogen, and silicon. Among them, halogenated flame retardants have been used less due to environmental safety issues, and metal hydroxides can reach effective flame retardance only with large additions. Compared with the above two flame retardants, phosphorus, nitrogen, and silicon compounds have been widely studied for their environmental friendliness and low addition. As a halogen-free flame retardant, intumescent flame retardants (IFRs) have the advantages of low smoke and low toxicity and have become the most widely used flame retardant [6,7,8]. There are three main substances in the common IFR system: acid source (the dehydration catalyst for char formation), carbon source (the carbonization agent), and foaming agent (the blowing agent). Ammonium polyphosphate (APP) is usually used as an acid source and a gas source to form protective carbon layers [9,10]. Although APP can provide satisfactory flame-retardant effects on many materials, as an additive flame retardant, it is easy to have poor compatibility with the materials matrix [11]. According to previous reports, adding less than 10 wt% APP helped TPU obtain a V-0 rate in a UL-94 test. Unfortunately, it lost more than 50% of its elasticity at the same time [12]. Therefore, it is a very challenging task to develop an efficient method to improve the flame-retardant efficiency of APP.

It is reported that transition metal compounds can promote carbon formation in polymers during combustion [13]. Adding a small amount of transition metal compound and combining with other flame retardants can effectively improve flame-retardant efficiency. Chen et al. [14] combined nickel citrate with APP to improve the flame-retardant properties of TPU, which significantly improved the graphitization degree of char residue of TPU. In addition, it has been found that copper ions (Cu^2+^) have a certain effect of catalyzing the formation of carbon, which can promotes flame-retardant efficiency. For example, Jiang et al. [15] used melamine formaldehyde resin to coat formaldehyde polyvinyl alcohol fiber and used Cu^2+^ to synergize flame retardancy. The addition of Cu^2+^ can effectively improve residual carbon and significantly improve the flame-retardant properties of the fiber. Therefore, we considered that Cu^2+^ can be used as a synergistic agent to improve the flame-retardant efficiency of APP.

Dopamine is a biological neurotransmitter, which can form polydopamine (PDA) by self-polymerization in alkaline aqueous solutions [16]. PDA is widely used for the surface modification of different materials because of its strong adhesion to various surfaces. In addition, PDA can be used as a char-forming agent to modify APP [17]. More importantly, the surface of PDA has a large number of functional groups such as amino and hydroxyl groups, which can provide abundant metal-chelating sites [18]. Cu^2+^-chelated PDA has a strong catalytic carbonization ability [5,19]. More dense carbon layers can be obtained to inhibit the transfer of heat and oxygen to the polymer, thereby effectively improving the flame-retardant properties of the polymer [20].

In order to endow TPU with good flame-retardant properties, we prepared APP-Cu@PDA by using two-step methods and then applied it to TPU to study the effect on the flame-retardant properties of TPU/APP-Cu@PDA composites. It was expected that APP-Cu@PDA would improve the flame retardancy of TPU. In addition, the catalytic carbonization ability of Cu^2+^-chelated PDA in TPU composites will be investigated.

## 2. Experimental Section

### 2.1. Materials

TPU and APP were obtained from Shandong Huada New Chemical Materials Co., Ltd. Dopamine hydrochlonride (DOPA, ≥98.0%) was supplied by Shanghai Macklin Biochemical Co., Ltd. Tris (hydroxymethyl) amino-methane (Tris) and Cu^2+^ chloride dihydrate (CuCl_2_·2H_2_O, ≥99.0%) were purchased from Tianjin Damao Chemical Reagent Factory.

### 2.2. Synthesis of APP-Cu

Generally, a certain proportion of ethanol and water (800:50 in volume) was transferred into a three-neck flask. APP measuring 10 and 0.845 g CuCl_2_·2H_2_O (5 mmol) were added into the flask, respectively, and stirred for 4 h. Subsequently, the solution was centrifuged again and rinsed with ethanol at least 3 times. It was then dried in a vacuum oven at 60 °C.

### 2.3. Synthesis of APP-Cu@PDA

**Scheme 1:**
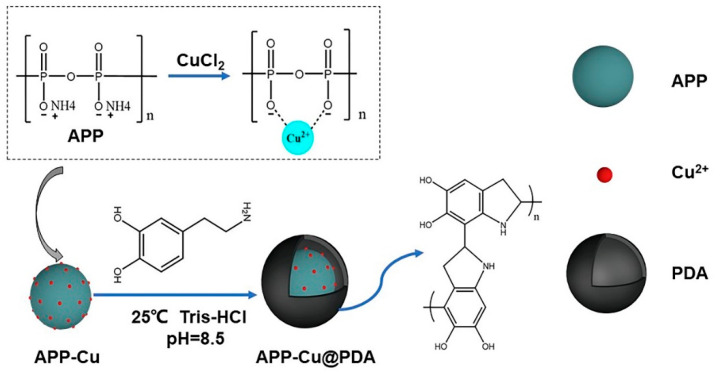
Scheme diagram of the preparation of APPCu@PDA.

The typical synthesis route of APP-Cu@PDA is provided in Scheme 1. First, APP-Cu (10 g) was added to 1000 mL of ethanol and sonicated for 30 min, and the above mixture was transferred into a three-necked flask. Tris 1.21 g measuring (10 mmol) and 1 g of dopamine (5 mmol) were dissolved in the mixture and stirred for 24 h. The reaction mixture was washed three times with ethanol, and the excess initial reactant was removed by centrifugation. Finally, the mixture was dried overnight in a vacuum oven at 60 °C to obtain APP-Cu@PDA particles.

### 2.4. Preparation of Flame-Retardant TPU Composites

The TPU were dried in a vacuum oven at 60 °C for 8 h. Then, they were mixed with different weight ratios of APP, APP-Cu, and APP-Cu@PDA, respectively, in an internal mixer, and the processing temperature was approximately 150 °C (Table 1). After mixing, the samples to be tested were compression-molded by using a hot-plate press at around 200 °C.

### 2.5. Measurements

Fourier transform infrared spectroscopy (FTIR) was used for recordings on a Nicolet MNGNA-IR560 (Artisan Technology Group, Austin, TX, USA) with a transition mode and a wave-number range between 400 cm^−^^1^ and 4000 cm^−^^1^. X-ray diffraction patterns (XRDs) were recorded on a D8 Advance X-ray diffractometer (Bruker, Karlsruhe, Germany) with Cu Kα radiation (λ = 0.154 nm). Thermogravimetric analysis (TGA) was recorded on an STA 449C thermal analyzer (Selb, Germany) with a heating rate of 10 °C/min in N_2_ atmosphere. X-ray photoelectron spectroscopy (XPS) was conducted by by using a VG ESCALAB MK II spectrometer with Al Kα X-ray radiation at 10 kV and 10 mA. A scan by scanning electron microscope (SEM) was carried out on SEM JEOL JSM-6360LV (Japan) equipped with mapping images. Limiting oxygen index (LOI) measurements were carried out according to ASTM D2863–13 on samples with dimensions of 127.0 mm × 6.5 mm × 3.0 mm. A UL-94 vertical burning test was performed on samples with dimensions of 127.0 mm × 12.7 mm × 3.0 mm according to ASTMD380. A cone calorimeter test (CCT) was used to characterize the combustion behavior of samples with dimensions of 100.0 mm × 100.0 mm × 4.0 mm under a heat flux of 50 kW/m^2^ according to ISO 5660-1. Raman spectra were obtained by using a multichannel confocal spectrometer (HORIBA Scientific LabRAM HR Evolution, Kyoto, Japan) with a laser wavelength of 535 nm.

## 3. Results and Discussion

### 3.1. Structure of APP-Cu@PDA

**Figure 1:**
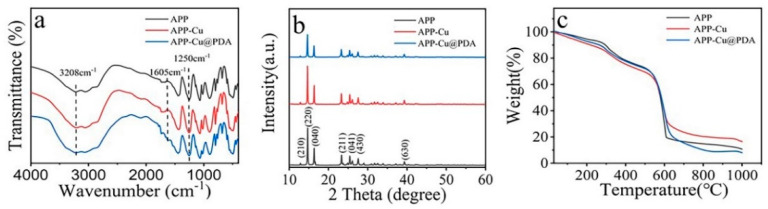
(**a**) FTIR spectra, (**b**) XRD patterns, and (**c**) TGA curves of APP, APP-Cu, and APP-Cu@PDA.

As shown in Figure 1a, the FTIR spectra of APP, APP-Cu, and APP-Cu@PDA showed adsorption peaks at 3208 cm^−1^ and 1250 cm^−1^, which is assigned to N-H and PO bonds. After being modified by PDA, several new absorption peaks appeared. The adsorption vibration peak of 1605 cm^−1^ was attributed to its indole group [21]. The appearance of the new peak confirmed that PDA was successfully coated on the surface of APP-Cu. TGA was used to further characterize the structure of APP-Cu@PDA. Figure 1b showed the XRD patterns of APP (PDF#45-0002), APP-Cu, and APP-Cu@PDA. It can be clearly observed that the introduction of Cu and PDA into APP had almost no effect on the crystal structure of APP. TGA was used to evaluate the thermal degradation behavior of APP, APP-Cu, and APP-Cu@PDA. It can be observed from Figure 1c that the thermal stability of APP-Cu@PDA was lower than that of APP, which may be due to the large number of unstable organic structures on PDA [22].

**Figure 2:**
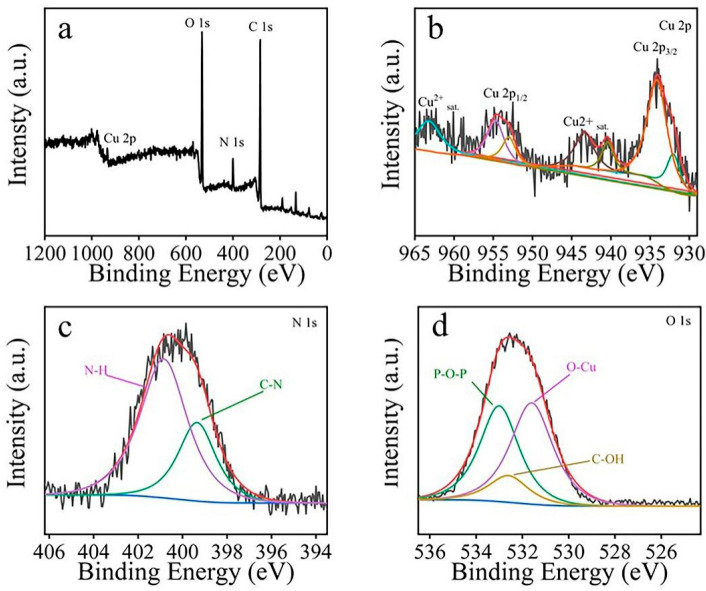
(**a**) XPS full spectra of APP-Cu@PDA, (**b**) Cu 2p, (**c**) N 1s, and (**d**) O 1s.

XPS was used to analyze the element types and chemical states of APP-Cu@PDA. Figure 2a showed the measured spectrum of APP-Cu@PDA, revealing the presence of Cu, N, O, and C. The Cu 2p spectrum (Figure 2b) can be divided into two peaks where the binding energies of 954.7 and 953 eV correspond to Cu 2p1/2 and 934.1 and 932 eV correspond to Cu 2p3/2 [23]. The two main intensities (Figure 2c) of 400.7 and 398.8 eV deconvoluted from the N 1s peak were attributed to N-H and C-N, respectively, [24] proving that there are abundant amino groups on the surface of APP-Cu@PDA, which may promote the interaction between the filler and the TPU matrix. [20] In addition, the deconvolution of the O 1s peak in Figure 2d showed P-O-P (533.1 eV), C-OH (532.6 eV), and O-Cu (531.6 eV) signals, which proved the presence of PDA and indicated the coordination bonds between Cu^2+^ and catechol groups [25].

**Figure 3:**
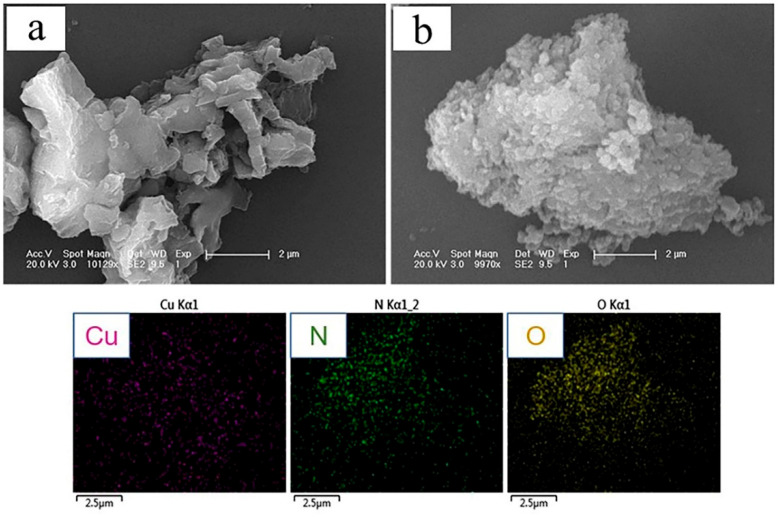
(**a**) SEM images of APP, (**b**) SEM images of APP-Cu@PDA, and mapping images of APP-Cu@PDA.

In order to further confirm PDA and Cu^2+^-modified APP, the obtained APP and APP-Cu@PDA materials were characterized by SEM to analyze their morphology and elemental composition. Figure 3a showed that the surface structure of APP was relatively smooth. After modification by Cu^2+^ and PDA, the surface became rough. The combination of SEM and mapping images of the selected area proved the existence of Cu^2+^, N, and O elements, indicating that Cu^2+^ and PDA were successfully coated on the surface of APP.

### 3.2. Thermal Degradation Behavior of TPU and TPU Composites

**Figure 4:**
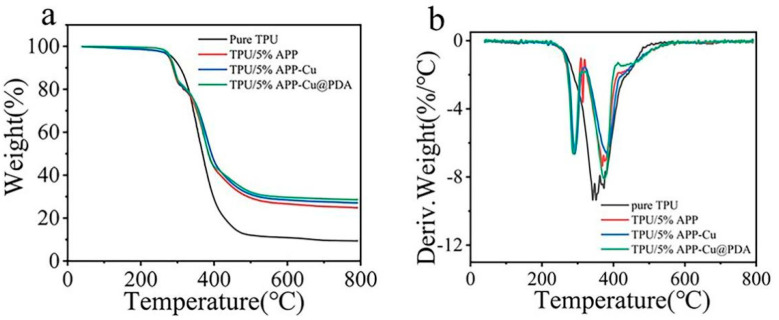
(**a**) TGA and (**b**) DTG curves of pure TPU and TPU composites under N_2_ at heating rate of 10 °C/min.

Under a nitrogen atmosphere, TGA and DTG were used to study the thermal stability of TPU and TPU composites (Figure 4), and the corresponding data were shown in Table 2. It can be observed from Table 2 that the initial degradation temperature 5 % weight loss of TPU/APP-Cu@PDA was lower than that of pure TPU. This may be due to the catalytic decomposition of APP-Cu@PDA and the introduction of a large number of organic functional groups on PDA [26]. It can be clearly observed from Figure 4 that the thermal stability of the composites with 5% APP had been significantly improved after 400 °C. The main reason was that the introduction of APP resulted in the formation of carbon layers at high temperatures, which inhibited the transfer of heat and oxygen to the substrate [27,28]. It was worth noting that char yields were 27.1% and 28.6% at 800 °C with the introduction of APP-Cu and APP-Cu@PDA, respectively, as compared with the pure TPU. It can be observed from the above results that the addition of PDA can improve the thermal stability of TPU composites. The main reason was that Cu^2+^-chelated PDA had a catalytic carbonization effect on the substrate, which improved the carbonization rate of TPU composites.

### 3.3. Combustion Behavior of TPU and TPU Composites

**Figure 5:**
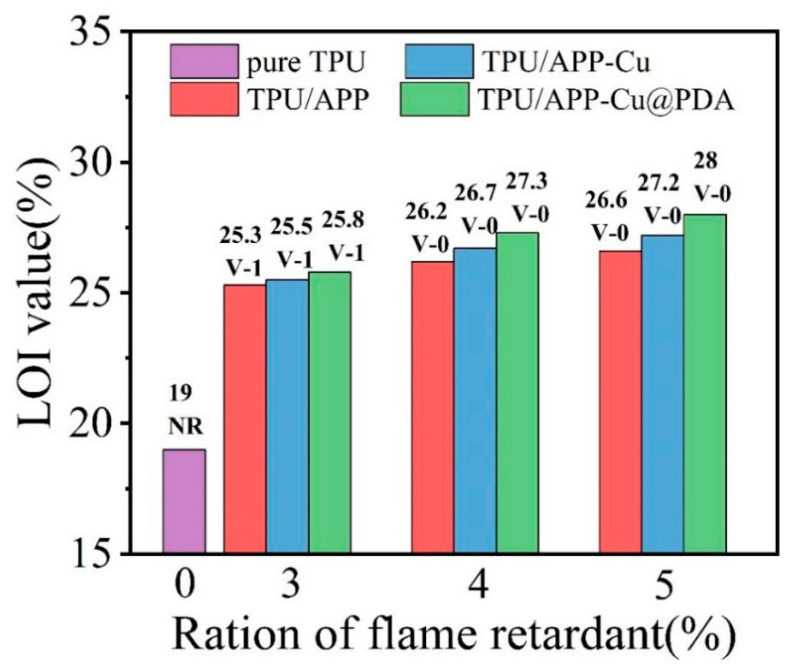
LOI and UL-94 results for TPU and TPU composites.

It can be observed from Figure 5 that the LOI of pure TPU was 19%. With the increase in flame-retardant loading, the LOI value gradually increases. In addition, the LOI value of adding different flame retardants (APP, APP-Cu, and APP-Cu@PDA) under the same addition amount was improved, and the LOI value of 5% APP-Cu@PDA can reach 28% higher than that of 5% APP (26.6%) and 5% APP-Cu (27.2%). The introduction of 5% APP-Cu@PDA increased the LOI value of composites by 9% compared with pure TPU. The higher LOI value of TPU/5%APP-Cu@PDA may be due to the higher catalytic ability of Cu^2+^-chelated PDA [20]. Therefore, the introduction of APP-Cu@PDA can generate relatively dense carbon layers to protect the polymer matrix, thereby achieving a better flame-retardant effect. In the UL-94 test, pure TPU burns violently and drips when ignited, failing to pass the V-0 rating. When the amount of flame retardant reaches 5%, the UL-94 rating is V-0. Moreover, TPU/5%APP-Cu@PDA stopped burning immediately after removing the flame, and the self-extinguishing ability was significantly improved.

**Figure 6:**
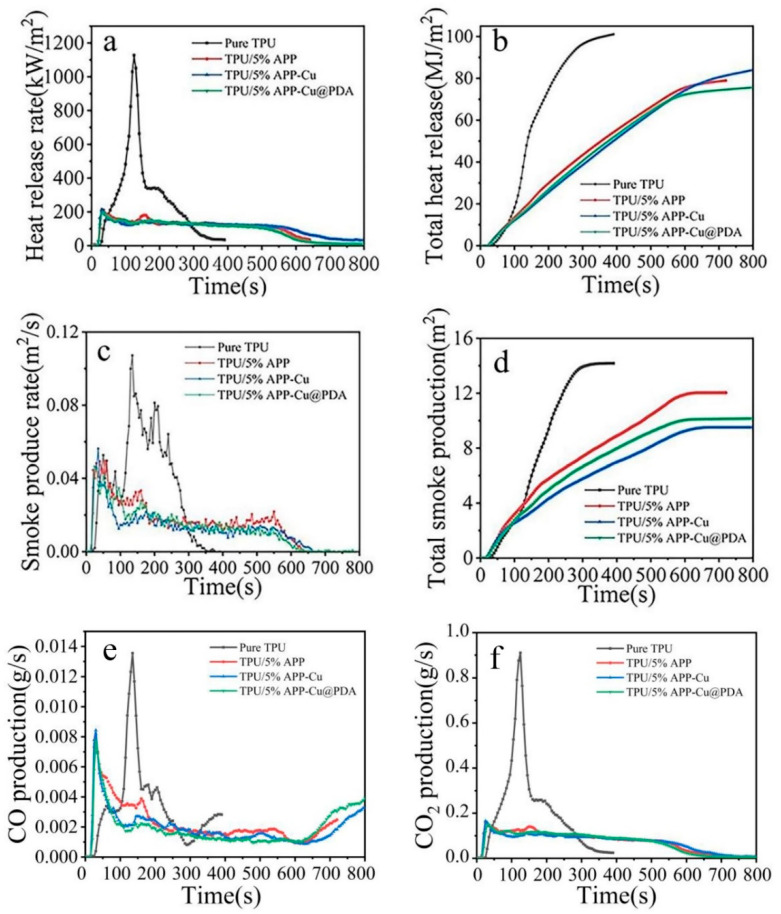
(**a**) HRR, (**b**) THR, (**c**) SPR, (**d**) TSP, (**e**) CO production, and (**f**) CO_2_ production curves of pure TPU and TPU composites.

In order to further study flame retardancy, CCT was carried out on pure TPU and TPU composites. The curves of heat release rate (HRR), total heat release (THR), smoke produce rate (SPR), total smoke produce (TSP), CO production, and CO_2_ production are shown in Figure 6 and Table 3. As shown in Figure 6a, pure TPU showed a sharp peak heat release rate (pHRR) at 1127 kW/m^2^ due to rapid combustion. For TPU/5%APP composites, pHRR had been reduced to 212 W/m^2^, mainly because APP can promote the dehydration and carbonization of polymers into carbon layers under thermal decomposition. After adding 5% APP-Cu@PDA, the pHRR of the composites further dropped to 196.3 kW/m^2^, which may be due to an increase in the formation of an expanded carbon layer [29]. The expanded carbon layer can act as a physical barrier to inhibit the transfer of heat and oxygen [22]. In addition, the THR of TPU/5%APP-Cu@PDA composite is lower than that of both TPU/5%APP and TPU/5%APP-Cu composites. The reason may be that the carbon layer generated was denser after adding PDA [29].

Additionally, the smoke produced during combustion has always been considered as an important factor that directly results in suffocation. Therefore, for flame-retardant polymers, smoke suppression is very important. Figure 6c showed the SPR curve of pure TPU and TPU composites. It can be observed that the peak of SPR (pSPR) of pure TPU was 0.11 m^2^/s. After adding 5% APP, APP-Cu6, and APP-Cu@PDA, the pSPR values of the composites were significantly reduced to 0.05 m^2^/s. This was mainly due to the formation of protective carbon layers on the surface of the polymer matrix, which was difficult to eliminate via the emitted gas. Figure 6d showed the TSP curve of pure TPU and TPU composites. It can be observed that the introduction of Cu^2+^ and Cu^2+^-chelated PDA can further reduce the TSP value of the TPU composites compared with pure TPU, respectively. However, the slightly higher TSP of TPU/5%APP-Cu@PDA composites is probably due to there being more organic functional groups on PDA, resulting in more gas released during the combustion process. In addition, compared with pure TPU, the CO and CO_2_ productions of TPU/5%APP-Cu@PDA composites were significantly reduced (Figure 6e,f). 

### 3.4. Char Layer Analysis

**Figure 7:**
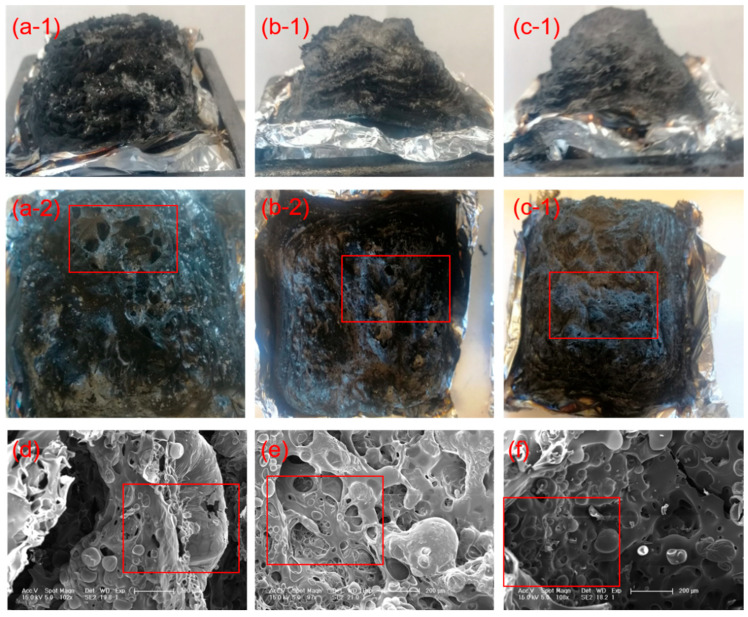
Digital images of chars from TPU/5%APP in top (**a-1**) and front (**a-2**) views, TPU/5%APP-Cu in top (**b-1**) and front (**b-2**) views and TPU/5%APP-Cu@PDA in top (**c-1**) and front (**c-2**) views, and SEM images of exterior chars from (**d**) TPU/5%APP, (**e**) TPU/5%APP-Cu, and (**f**) TPU/5%APP-Cu@PDA.

In order to further analyze the charring ability of APP-Cu@PDA after combustion, digital images of the outer surface of the carbon residue tested by a cone calorimeter were taken. As shown in Figure 7a, after adding 5% of APP, carbon residues were measured to be lower and the carbon layers on the outer surface were relatively fragile; thus, there were many large and dense holes on the top. After adding 5% APP-Cu (Figure 7b) and 5% of APP-Cu@PDA (Figure 7c), the amount of residual carbon increased significantly, and the holes in the outer surface carbon layer became smaller, which can inhibit the transfer of heat and oxygen. In addition, SEM images were used to observe the changes in the microstructure of carbon residues of different TPU composites. It can also be observed in Figure 7d–f that the carbon layers of the TPU composites with 5% of APP-Cu@PDA were mostly intact and compact, which further illustrates the catalytic carbonization ability of Cu^2+^-chelated PDA on TPU materials.

**Figure 8:**
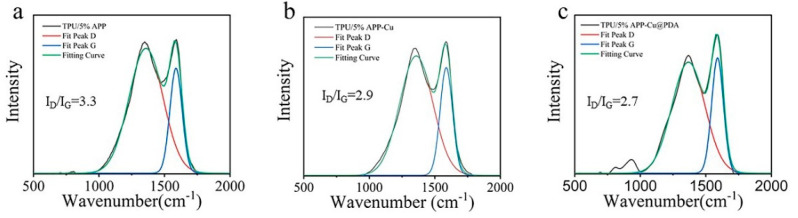
Raman spectra of residues for (**a**) TPU/5%APP, (**b**) TPU/5%APP-Cu, and (**c**) TPU/5%APP-Cu@PDA.

In addition, the resulting carbon residue after combustion was analyzed by Raman spectra (Figure 8). The Raman spectrum showed two representative peaks at 1348 and 1590 cm^−^^1^, which belong to peak D and peak G, respectively. The ratio of D peak intensity to G peak intensity was used to judge the degree of graphitization of the carbon residue. The lower the value of I_D_/I_G_, the higher the degree of graphitization. The I_D_/I_G_ values of TPU/5%APP, TPU/5%APP-Cu, and TPU/5%APP-Cu@PDA were 3.3, 2.9, and 2.7, respectively. It was proved that TPU/5%APP-Cu@PDA had the highest degree of graphitization [19].

### 3.5. Mechanical Properties

The mechanical properties of pure TPU and TPU composites are shown in Table 4. The results showed that the tensile strength (T_s_) of pure TPU was 28.9 ± 2.3 MPa, and elongation at break (E_b_) was 1310 ± 42%. The introduction of APP significantly reduced the T_s_ and E_b_ of TPU composites, which may be due to the stress concentration caused by the accumulation of APP in the TPU matrix, making the composites prone to cracks [30]. Furthermore, compared with TPU/5%APP, the T_s_ and E_b_ of TPU/5%APP-Cu increased by 25% and 12%, and the T_s_ and E_b_ of TPU/5%APP-Cu@PDA increased by 43% and 37%. This may be due to the rigidity of the APP-Cu component and the strong interaction between the PDA and the TPU matrix in the APP-Cu@PDA component, which further improved compatibility with the matrix [20].

### 3.6. Proposed Fire-Retardant Mechanism

**Scheme 2:**
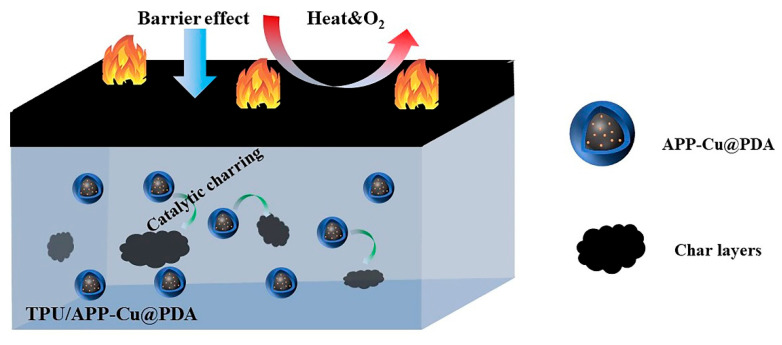
Schematic diagram of the flame-retardant mechanism of TPU/APP-Cu@PDA composite.

Based on the above results and analysis, we proposed the flame-retardant mechanism of APP-Cu@PDA in TPU composites shown in Scheme 2. Cu^2+^-chelated PDA had the ability to catalyze the carbonization of TPU in the condensed phase, making carbon layers denser. Moreover, dense carbon layers can act as a barrier, reducing the transfer of heat, fuel, and oxygen between the flame zone and the polymer matrix. The higher the strength of the carbon layer, the better the flame retardancy.

In addition, compared with other reported flame-retardant TPU composites (Table 5), the bio-based TPU/APP-Cu@PDA composites prepared in this paper use a simple and green synthetic route and have excellent flame-retardant effects. Therefore, the material is expected to be used as an environmentally friendly and efficient flame retardant in the industrial production of fireproof materials in the future.

## 4. Conclusions

In order to provide TPU with higher flame-retardant properties, bioinspired Cu^2+^-chelated PDA was used to coat the surface of APP to prepare TPU composites. The successful preparation of TPU/APP-Cu@PDA was proved by FTIR, XRD, XPS, and SEM analysis. In terms of flame-retardant testing, the LOI value of pure TPU was 19%, there were no ratings in UL-94, and pHRR was 1127.3 kW/m^2^. Compared with pure TPU, the LOI value of TPU/5%APP-Cu@PDA was 28%, and the V-0 rating and pHRR were reduced to 196 kW/m^2^. The introduction of Cu^2+^ and PDA can further improve the flame-retardant efficiency of APP catalyzing carbonization. The SEM and Raman studies on residual carbon showed that Cu^2+^-chelated PDA improves the quality of carbon and has a higher degree of graphitization. These results indicated that APP-Cu@PDA can form dense carbon layers, which act as a physical barrier and inhibit the transfer of heat and oxygen. It was worth noting that APP-Cu@PDA is a non-toxic, smoke-suppressing, and highly effective flame retardant, and Cu^2+^-chelated PDA can be used to modify the surface of various other fillers, providing a method for the preparation of high-performance flame-retardant polymer composites.

## Figures and Tables

**Table 1:** The formulations of TPU composites.

Sample Code	TPU/g	APP/g	APP-Cu/g	APP-Cu@PDA/g
Pure TPU	100	/	/	/
TPU/3%APP	97	3	/	/
TPU/4%APP	96	4	/	/
TPU/5%APP	95	5	/	/
TPU/3%APP-Cu	97	/	3	/
TPU/4%APP-Cu	96	/	4	/
TPU/5%APP-Cu	95	/	5	/
TPU/3%APP-Cu@PDA	97	/	/	3
TPU/4%APP-Cu@PDA	96	/	/	4
TPU/5%APP-Cu@PDA	95	/	/	5

**Table 2:** TGA data for TPU and TPU composites.

Sample	T_5wt%_ (°C)	T_max_ (°C)	C_800_ (%)
Pure TPU	285	351	9.4
TPU/5% APP	276	369	24.8
TPU/5%APP-Cu	277	379	27.1
TPU/5%APP-Cu@PDA	279	373	28.6

Note: T_5wt%_ means temperature at 5 wt% mass weight, T_max_ means temperature at the maximum degradation rate, and C_800_ means char yield at 800 °C.

**Table 3:** CCT data for TPU and TPU composites.

Sample	TTI (s)	pHRR (kW/m^2^)	THR (MJ/m^2^)	pSPR (m^2^/s)	TSP (m^2^)	FPI (s m^2^/kW)	Residue (%)
Pure TPU	27	1127	101.1	0.11	14.2	0.024	8.5
TPU/5%APP	21	212	77.2	0.05	12.0	0.099	18.3
TPU/5%APP-Cu	22	227	84.0	0.05	9.5	0.097	22.8
TPU/5%APP-Cu@PDA	25	196	75.7	0.05	10.1	0.127	23.0

**Table 4:** Tensile tests for pure TPU and TPU composites.

Sample	T_s_ (MPa)	E_b_ (%)
Pure TPU	28.9 ± 2.3	1310 ± 42
TPU/5%APP	20.0 ± 0.6	991 ± 24
TPU/5%APP-Cu	25.3 ± 0.3	1127 ± 71
TPU/5%APP-Cu@PDA	28.6 ± 0.4	1361 ± 5

**Table 5:** Flame-retardant properties of TPU composites in the literature.

Year	Sample	Bio-Based Supporter	FR	LOI Increase (%)	pHRR Decrease (%)	Ref.
2018	TPU/para-aramid fiber-Fe	NO	Para-aramid fiber-Fe	11.8	57.6	[31]
2019	TPU/CS	YES	CS	-	65.9	[32]
2019	TPU/oyster shell powder/isopropyl tris(dioctylphosphoryloxy) titanate	NO	isopropyl tris(dioctylphosphoryloxy) titanate	6.5	67.5	[33]
2021	TPU/SiAPP/MPCSN	NO	SiAPP/MPCSN	29	68.7	[34]
2021	TPU/Zn&Co-ZIF-L	NO	Zn&Co-ZIF-L	9.7	43.8	[35]
2022	TPU/8.0AHP/1.0ZIF-8@SEP	NO	ZIF-8@SEP	20.3	78.9	[36]
2022	TPU/APP-Cu@PDA	YES	APP-Cu@PDA	32.1	82.0	This work

## Data Availability

Data is contained within the article.
